# Potential Trends of Point-of-Care Diagnostics—The Next Generation of the Laboratory Diagnosis

**DOI:** 10.3390/diagnostics10100774

**Published:** 2020-09-30

**Authors:** Sheng-Wen Lin, Ching-Fen Shen, Chao-Min Cheng

**Affiliations:** 1 Institute of Biomedical Engineering, National Tsing Hua University, Hsinchu 300, Taiwan; wenwenlintw@gmail.com; 2 Department of Pediatrics, National Cheng Kung University Hospital, College of Medicine, National Cheng Kung University, Tainan 704, Taiwan

With the current worldwide outbreak of COVID-19, developing rapid, effective, and convenient detection tools has become imperative. While developing such detection tools for COVID-19 is a highly critical endeavor, similar endeavors have and continue to be undertaken to develop better diagnostic and detection tools for a variety of other diseases as well. Most current laboratory diagnostics are complex and time/labor intensive. However, frequent and timely testing via point-of-care (POC) diagnostics is highly desirable because patient health status can change rapidly, early detection facilitates early intervention, and follow-up care requires recurrent diagnostic examination. All of these efforts improve the level of care and promote positive health outcomes. Based on current global medical trends, POC diagnostic kits could be used at the bedside to provide rapid, preliminary results and impart tremendous impact on the medical ecosystem.

## 1. Image Analysis and Liquid Biopsy

Current diagnostic methods for examining male infertility primarily rely on microscope-based examination and computer-assisted semen analysis (CASA) systems to process and examine human semen [1]. Although standard methods could provide a holistic approach for determining sperm quality, such approaches require bulky and costly microscopes, equipment, and well-trained technicians. Moreover, these requirements are complicated, expensive, often costing hundreds of US dollars, and time intensive. They also limit accessibility to semen analysis in resource-limited areas and push the process into the category of non-routine health examinations.

**Figure 1:**
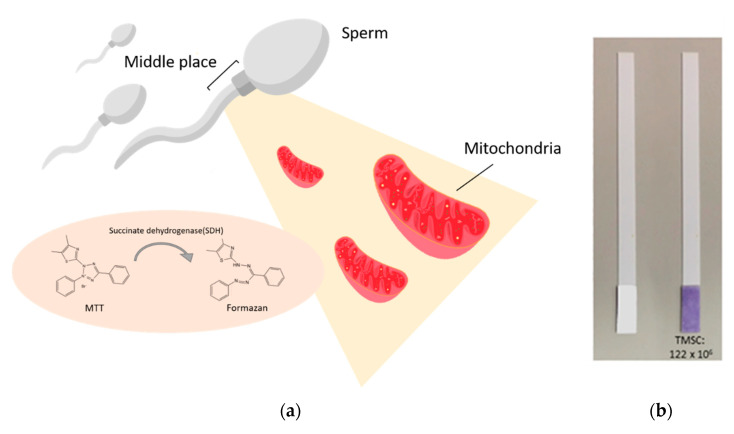
The mechanism and colorimetric results from a 3-(4,5-Dimethylthiazol-2-yl)-2,5-diphenyltetrazolium bromide (MTT) test strip. (**a**) The mechanism of sperm and MTT interaction. There is an abundance of mitochondria in the middle portion of the sperm cell, where succinate dehydrogenase (SDH) located on the mitochondria transforms MTT into formazan. (**b**) The color of the MTT test strip before and after testing the semen sample. The colorimetric result is homogenously distributed over the testing zone of the MTT test strip and the result can be easily interpreted by visual observation or with a smartphone-based recording and analytical system. MTT: 3-(4,5-Dimethyl-2-thiazolyl)-2,5-diphenyl-2H-tetrazolium bromide. (Adapted image from [2]).

To meet the need for a fast, convenient, and easy-to-operate semen analysis tool, biochemical analysis might be a suitable diagnostic approach. A novel, extremely inexpensive, and easy-to-use alternative to conventional methodology can be found in the form of a paper-based diagnostic device capable of detecting total motile sperm concentration (TMSC) based on the mitochondrial activity of motile spermatozoa [2] (Figure 1). This tool can be used to distinguish low TMSC semen samples from those with normal TMSC levels. The activity and function of mitochondria are key to sperm function and play a key role in many sperm functions including basic motility, acrosome reaction, and final fertilization [3]. Moreover, the activity of succinate dehydrogenase (SDH), one of the mitochondrial respiratory chain enzymes, was found to be highly correlated with sperm quality, including sperm concentration, motility, and vitality [4]. This paper-based diagnostic device is used to assess mitochondrial activity of sperm based on 3-(4,5-Dimethylthiazol-2-yl)-2,5-diphenyltetrazolium bromide (MTT). MTT is a yellow tetrazolium salt that can be reduced by SDH, in the mitochondria of metabolically active cells, to purple-colored formazan. The amount of formazan is proportional to the metabolic activity of living cells, suggesting that MTT can be used as a powerful tool for providing basic information about sperm quality.

Compared with image detection, liquid biopsy could be more convenient and practical. It is not limited by location, instrument, time, or personnel. Although liquid biopsy is relatively less likely to provide accurate results compared to image detection, it may be used to help doctors obtain a rapid, preliminary result in the clinic or at bedside. Results could be used to expedite follow-up care protocols. Similar biochemical analysis tools can be developed to examine urine, tears, sweat, etc. These simple and easy-to-use diagnostic kits will be able to help clinicians and individuals obtain results more quickly and effectively.

## 2. Bedside Point-of-Care (POC) Diagnostics Using Specific Biomarkers

Bacterial and viral infections are the most common reasons children receive medical care in clinics or small hospitals. As patients with bacterial and viral infections exhibit similar symptoms, symptom-based tests or a medical history review are often insufficient for distinguishing between bacterial and viral infections, even among experienced doctors. Although treatment options for bacterial and viral infections differ substantially, children are often treated with empirical antibiotics because of the lack of rapid and accurate testing. This can lead to delayed diagnosis and antibiotic abuse, increasing the incidence of adverse events due to antibiotics, antibiotic resistance, and other potential confounding issues.

Current biochemical analyses based on biomarkers, such as procalcitonin (PCT) and C-reactive protein (CRP) methods, can provide some clues for the differential diagnosis of bacterial and viral infections, but their low sensitivity and specificity means that they cannot be relied upon in and of themselves for treatment. Moreover, bacteria-virus interactions and their relationship to biofilm formation make pathogen diagnosis more challenging. Both of these issues underscore the need for the development and use of POC diagnostics using host biomarkers to distinguish between bacterial and viral infections in pediatric patients. Such an approach allows for more careful selection of appropriate therapies.

**Figure 2:**
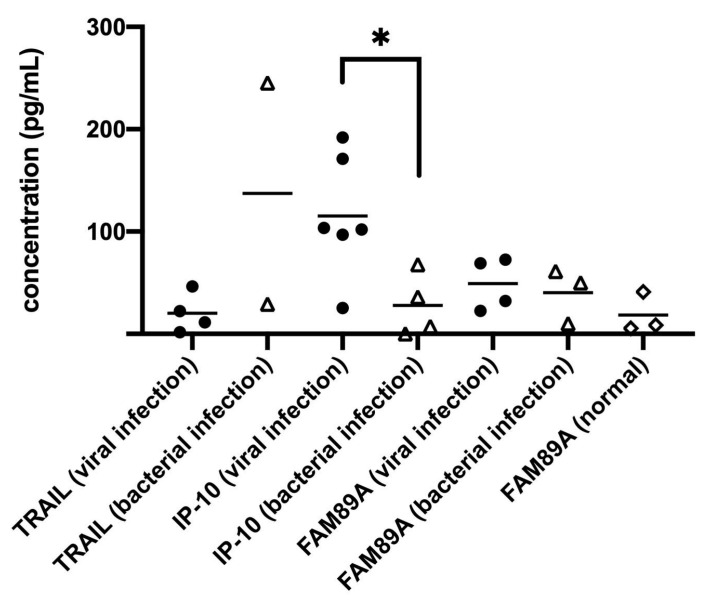
Several biomarkers applied to detect infection modes in children, including TNF (Tumor necrosis factor)-related apoptosis-inducing ligand (TRAIL), interferon γ-induced protein 10 (IP-10) and family with sequence similarity 89 member A (FAM89A). The symbols (●△◇) in this figure mean different patient samples in different study groups. The IP-10 concentration in urine demonstrated a significant difference between viral and bacterial infections (* *p* < 0.05).

We have discussed the use of several biomarkers, including TNF (Tumor necrosis factor)-related apoptosis-inducing ligand (TRAIL), interferon γ-induced protein 10 (IP-10) and family with sequence similarity 89 member A (FAM89A) to analyze the urine of children. We preliminarily found a significant difference in the concentration of IP-10 among children with viral infections compared to children with bacterial infections (Figure 2). From our preliminary data, it can be known that IP-10 in urine could be used to differentiate the infection mode, viral or bacterial, in children, and these data can potentially be used to guide clinical treatment, once its sensitivity and specificity in urine are defined.

The development and discovery of new biomarkers to gauge health status is ongoing. Several biomarkers have recently demonstrated potential value for use as bacterial infection markers, including IL-27, CD35, CD64, and presepsin [5,6,7]. While most studies have only detected biomarkers in blood samples, non-invasive samples such as nasopharyngeal aspirates, saliva, or urine may also be used to detect infection. Non-invasive sample analysis provides several advantages; it is painless, enables self-collection, and offers superior safety for both patient and doctor. However, the use of non-invasive samples, such as urine and saliva, is usually limited by fluctuations in sample flow. This directly affects the sensitivity and specificity of detection. If this problem can be overcome, analysis of biomarkers within non-invasive samples could become a valuable approach for diagnosis and subsequent treatment of infants and children and will facilitate the development of easy-to-use POC.

As the number of confirmed cases of COVID-19 increases rapidly around the world, we know that conventional testing may become insufficient. We need more efficient, rapid diagnostic methods to maintain a sustainable medical ecosystem. In the future, we expect that testing by in non-invasive samples and the development of new biomarkers will lead to more reliable and convenient testing methods that will reduce transport requirements, the risk of infection transmission, stress on healthcare systems, and healthcare costs for both individuals and governments.
